# Amphiphilic Celecoxib-Polysaccharide Delivery System for Enhanced Colon-Targeted Colitis Therapy

**DOI:** 10.3390/pharmaceutics17040511

**Published:** 2025-04-12

**Authors:** Qiao Qiao, Xian Wan, Jie Li, Weijun Chen, Enxuan Li, Lipeng Qiu, Huiming Tu

**Affiliations:** 1 Department of Gastroenterology, Affiliated Hospital of Jiangnan University, Wuxi 214122, China; bojuejojo@163.com; 2 School of Life Sciences and Health Engineering, Jiangnan University, Wuxi 214122, China; 6233304014@stu.jiangnan.edu.cn (X.W.); 6211507007@stu.jiangnan.edu.cn (J.L.); wjcaiwhl@jiangnan.edu.cn (W.C.); 3 The Second Clinical Medical School, Nanjing Medical University, Nanjing 210029, China

**Keywords:** celecoxib, chondroitin sulfate, drug delivery, ulcerative colitis

## Abstract

**Background**: Ulcerative colitis (UC), a subtype of chronic inflammatory bowel disease (IBD), is primarily treated with oral medications to reduce inflammation and alleviate symptoms. Celecoxib (CXB) is an attractive candidate for UC; however, its limited solubility and low bioavailability pose significant challenges to its clinical application. **Methods**: We reported a novel chondroitin sulfate A–Celecoxib (CSA-CXB) polymeric nanoprodrug to address the limited solubility and low bioavailability of CXB. CXB was conjugated to chondroitin sulfate A (CSA) via succinic anhydride (SA) and ethylenediamine to prepare CSA-CXB polymers, which can self-assemble into nanoparticle structural prodrugs in aqueous condition. We investigated the stability, blood compatibility, and responsiveness of the nanoparticles. The ability of the nanoparticles to treat UC in vitro and in vivo was then evaluated. **Results**: The CSA-CXB nanoprodrug was spherical with a mean particle size of 188.4 ± 2.2 nm, a zeta potential of −22.9 ± 0.1 mV, and sustained drug release behavior. Furthermore, CSA-CXB exhibited remarkable antioxidant and anti-inflammatory effects, as it can significantly increase the free radical scavenging rate and reduce the expression level of ROS, TNF-α, IL-6, nitric oxide (NO), and COX-2 protein in vitro. In vivo results demonstrated that CSA-CXB targeted the mice’s colon efficiently mitigate UC symptoms by inhibiting the expression of inflammatory cytokine. **Conclusions**: The CSA-CXB nanoprodrug can improve the therapeutic impact of CXB, and has potential as a new preparation for a clinical UC treatment nanoprodrug.

## 1. Introduction

Ulcerative colitis (UC) is an enduring inflammatory bowel disorder that usually presents as periodic inflammatory episodes in the colon. During the recent decades, the worldwide incidence of UC has significantly risen, and approximately 5 million people worldwide will be affected by UC in 2023 [1,2]. UC is difficult to treat and necessitates lifelong medical management, thus, the patient’s quality of life suffers. The degree of damage caused by UC can vary from mild to severe, with symptoms including persistent diarrhea, abdominal pain, and weight loss. In severe cases, UC may progress to life-threatening complications such as rectal bleeding and colorectal cancer [3,4,5]. The pathogenesis of UC is multifaceted with no definitive conclusion. The widely accepted view is that dysregulated immune response, altered gut microbiota, and defective gut epithelial barrier play a significant part in the development of UC [6]. Multiple factors contribute to the disruption of intestinal barrier function, permitting more microbiota to cross the barrier, and thereby activating the immune response in colonic lesions, which results in the escalated release of pro-inflammatory cytokines. Meanwhile, the inflamed mucosa is infiltrated by activated macrophages and granulocytes, whose subsequent release of large amounts of NO may cause sustained tissue damage [7]. Inflammation also promotes reactive oxygen species (ROS) production and inhibits ROS scavenging. Consequently, this ROS accumulation induces oxidative stress damage to intestinal epithelial cells.

At present, UC is treated primarily with pharmaceuticals (including corticosteroids, biologics, and 5-aminosalicylic acid analogs) for achieving recession or maintenance to prevent long-term complications, or in some cases, with surgical revision or removal of the diseased portion of the gastrointestinal tract [8]. Among them, general anti-inflammatory agents remain the first choice [9]. As a non-steroidal anti-inflammatory drug (NSAID), CXB blocks prostaglandin production by selectively inhibiting cyclooxygenase-2 (COX-2), resulting in anti-inflammatory and analgesic effects. CXB is generally used to relieve the symptoms of osteoarthritis, rheumatoid arthritis, and ankylosing spondylitis [10]. It is reported that CXB can improve UC by specifically inhibiting COX-2 [11]. Additionally, studies have shown that CXB enhances the intestinal barrier and alleviates ferroptosis and apoptosis signaling pathway and, hence, alleviated symptoms in a UC mouse model [12]. However, poor water solubility (3–7 μg/mL, BCS Class 2) and non-targeting limit its therapeutic efficacy [13]. It can also carry risks, including renal and liver toxicity, anaphylaxis, and Stevens–Johnson syndrome [14,15]. Therefore, it is urgent to find a new strategy to deliver CXB for UC therapy.

Polysaccharide polymers are frequently utilized in pharmaceutical delivery systems given their favorable biocompatibility and easy biodegradation [16]. The numerous reactive groups on the polysaccharide chain make it easy to couple chemically [17,18]. Polysaccharide polymers and drug molecules are chemically coupled through covalent bonds to form polymer–drug conjugates (PDCs), thus prolonging the in vivo residence and improving solubility [19,20]. Chondroitin sulfate (CS) is a natural glycosaminoglycan (GAG) with high water solubility and multiple pharmacological effects. Previous studies have shown that CS conjugation with insoluble drug molecules could improve solubility [21]. Importantly, CS has shown immunomodulatory and anti-inflammatory activities by suppressing the synthesis of pro-inflammatory cytokines and pro-inflammatory enzymes [22,23]. Additionally, CS exhibits a selective binding affinity towards a cluster of differentiation 44 (CD44) [24]. CD44 is a transmembrane glycoprotein, abundantly present on the surface of macrophages and colonic epithelial cells within inflammatory tissues [25,26]. Recently, CS-based nanoparticles were successfully applied for CD44-mediated targeted drug delivery in ulcerative colitis [27,28,29,30,31]. Compared to the normal tissues, the inflammatory intestine presented increased vascular permeability. This increase makes it easier for nanoparticles to leak out of the blood vessels and accumulate in the inflamed areas after administration; this phenomenon is commonly referred to as the enhanced permeability and retention effect [32,33,34]. Therefore, nanoscale drug delivery systems are good choices for the treatment of UC. 

In this study, a delivery system based on a chondroitin sulfate A–celecoxib (CSA-CXB nanoprodrug) polymeric nanoprodrug was prepared for UC therapy. This new nano delivery system does not only enhance the water solubility and targeting of CXB but also has a synergistic effect of polymeric carriers and drugs in the inflamed area. The CSA-CXB nanoprodrug was synthesized by modifying CXB onto CSA backbone via condensation reactions of amine and carboxylic acid. Subsequently, CSA-CXB was characterized for its structure and pharmaceutical properties, and then in vivo and in vitro anti-inflammatory effects were explored in lipopolysaccharide (LPS)-stimulated RAW264.7 cells and dextran sulfate sodium salt (DSS)-induced UC mice. The nanoprodrug exhibited increased anti-inflammatory and antioxidant properties both in vivo and in vitro, alleviating the symptoms of UC.

## 2. Materials and Methods

### 2.1. Materials

CXB (crystalline Form III) was purchased from Energy Chemical Co., Ltd. (Shanghai, China). CSA (11 kDa), (1-ethyl-3-(3-dimethylaminopropyl)) carbodiimide hydrochloride (EDC·HCl), and *N*-Hydroxy succinimide (NHS) were purchased from Aladdin Biochemical Technology Co., Ltd. (Shanghai, China). SA and ethylenediamine were purchased from Sinopharm Chemical Reagent Co., Ltd. (Shanghai, China). The NO kit was purchased from Shanghai Beyotime Biotech Co., Ltd. (Shanghai, China). ELISA kits were purchased from Thermo Fisher Scientific (Shanghai, China). The 1,1-diphenyl-2-picrylhydrazyl radical (DPPH) was purchased from Shanghai Macklin Biochemical Co., Ltd. (Shanghai, China).

### 2.2. Synthesis and Characterization of CSA-CXB Polymers

**Figure 1:**
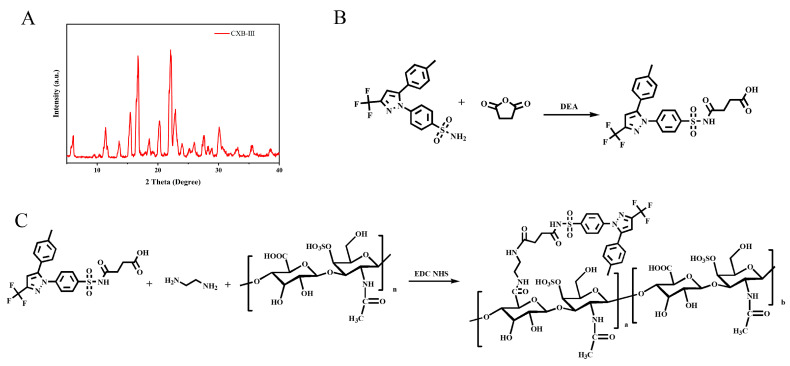
The PXRD pattern of CXB (**A**). The synthetic routes of CXB-SA (**B**) and CSA-CXB (**C**).

Celecoxib succinate (CXB-SA) was first synthesized as shown in Figure 1A, according to the published literature [35]. In brief, 0.381 g of CXB, 0.151 g of SA, and 0.285 mL of Triethylamine (TEA) were stirred under reflux with 5 mL Dichloromethane (DCM) at 60 °C for 12 h. After being purified with column chromatography (CH_2_Cl_2_/MeOH = 50/1), the solution was evaporated at 60 °C and subjected to vacuum drying to yield the CXB-SA solid.

CXB-SA was linked to CSA through EDC/NHS-mediated condensation reactions of amine and carboxylic acid with ethylenediamine (Figure 1B). Then, 48.1 mg of CXB-SA and 48.9 mg of CSA were dissolved in a 5 mL formamide solution. The carboxyl group was activated by EDC (38.3 mg) and NHS (34.5 mg), and then, 10 µL of ethylenediamine was added. After full reaction, the unreacted substrate was removed by dialysis (MWCO = 3000 kDa), and then freeze-dried to obtain CSA-CXB polymers.

CXB-SA and CSA-CXB were characterized by Fourier-transform infrared spectroscopy (FTIR, Nicolet, Bicester, UK) with the following parameters: spectral range: 4000–400 cm^−1^; resolution: 4 cm^−1^; scan number: 32 scans per spectrum. The ^1^H nuclear magnetic resonance spectra (NMR, Bruker, Billerica, MA, USA) were also used to measure CXB-SA and CSA-CXB with a frequency of 400 MHz.

### 2.3. Preparation and Characterization of CSA-CXB Nanoprodrug

The CSA-CXB polymers dissolved in aqueous solution (1 mg/mL) were subjected to ultrasonication, followed by filtration through a 0.45 μm membrane filter, yielding CXB-CSA nanoprodrug suspension for further use. Dynamic light scattering (DLS, Malvern Instruments, Malvern, UK) was used to measure the particle size and zeta potential. Morphological observations were measured through transmission electron microscopy (TEM, JEOL, Tokyo, Japan).

The stability of the CSA-CXB nanoprodrug in blood was investigated by monitoring particle size change. Briefly, the CSA-CXB nanoprodrug was mixed with 20% FBS and incubated in a shaker. The particle size of the nanoprodrug was determined at specific time points.

### 2.4. In Vitro Drug Release Test

The in vitro drug release of CXB from the CSA-CXB nanoprodrug was investigated according to a previously reported method [36]. CSA-CXB solution (2 mg/mL) was transferred into dialysis bags, which were incubated at 37 °C under 100 rpm shaking with 10 mL PBS (pH 7.4). At pre-set time intervals (0, 0.5, 1, 2, 4, 6, 8, 10, 12, 24, 48, 72 h), 1 mL of release medium was removed, then replaced with an equal amount of fresh PBS. 

The cumulative release of CXB was quantified using a high-performance liquid chromatography (HPLC) system under the following chromatographic conditions: Column: Agilent TC-C18 analytical column (250 mm × 4.6 mm inner diameter, 5 μm particle size). Mobile phase: A mixture of methanol and buffer solution (0.27% (*w*/*v*) potassium dihydrogen phosphate, pH adjusted to 3.0 with phosphoric acid) in a ratio of 65:35 (*v*/*v*). Detection wavelength: 254 nm. Flow rate: 1.0 mL/min. Column temperature: Maintained at 30 °C using a thermostatically controlled column oven. Injection volume: 10 µL. The cumulative drug release (%) is calculated as follows:

$$\begin{array}{c} Er\left(\%\right)=\frac{V_{e}\sum_{1}^{i-1}C_{i}+V_{0}C_{i}}{m_{D}}\times 100\% \end{array}$$

where Er represents the cumulative drug release, V_e_ is the volume of release medium per removal, C_i_ is the sample drug concentration at the i-th sampling time, V_0_ is the total volume of the release medium, and m_D_ is the total mass of CXB in the CSA-CXB nanoprodrug.

### 2.5. In Vitro Antioxidant Capability Study

Ethanol solutions of DPPH (0.1 mM, 2 mL) were incubated, respectively, with CSA-CXB, free CSA, free CXB, and CSA + CXB (200 μL) under dark conditions at room temperature for 30 min. Each sample’s absorbance was assessed at 517 nm. H_2_O_2_ solution (10 mM, 2 mL) was incubated with the same sample for 10 min to measure the scavenging activity of H_2_O_2_, and at a wavelength of 230 nm, the absorbance was evaluated. The scavenging activity effect (%) can be determined as follows:

$$\begin{array}{c} Scavenging\;effect\;(\%)=(1-\frac{A_{1}}{A_{0}})\times 100\% \end{array}$$

where A_0_ is the absorbance value of the DPPH or H_2_O_2_ solution, and A_1_ is the absorbance value of the mixed solution of samples.

### 2.6. Hemolysis Test

The mice red blood cell suspension (5%) was prepared from C57BL/6 mice’s blood and subsequently incubated with the same volume of different concentrations of CSA-CXB (50–1000 μg/mL) at 37 °C for 2 h. Subsequently, the mixture was centrifuged at 3000 rpm for a duration of 5 min. A full-wavelength scan using a UV spectrophotometer was performed on the upper solution. Water was utilized as a positive (100% hemolysis) control, while PBS was used as a negative (0% hemolysis) control. The hemolysis rate was determined as follows:

$$\begin{array}{c} Hemolysis\left(\%\right)=\frac{A\;-{\;A}_{0\%}}{A_{100\%}-A_{0\%}}\times 100\% \end{array}$$

where A, A_0%_, and A_100%_ are the absorbance values of the sample, 0% hemolysis, and 100% hemolysis, respectively.

### 2.7. In Vitro Cytotoxicity

RAW 264.7 (mouse mononuclear macrophages) and 3T3 cells (mouse embryonic fibroblast cells) were collected from the Collection Center of the Chinese Academy of Sciences (Shanghai, China). The cytotoxicity of CSA-CXB was investigated by Methylthiazolyldiphenyl-tetrazolium bromide (MTT) assay. RAW264.7 and 3T3 cells were inoculated to allow attachment. Subsequently, the culture medium was aspirated from each well and the medium containing CSA-CXB was added. MTT solution was added after 24 h of incubation, then the medium was removed, and dimethyl sulfoxide (DMSO) was added 4 h later. The absorbance at 570 nm was subsequently quantified using a microplate reader. Cell viability was calculated as follows:

$$\begin{array}{c} Cell\;Viability\left(\%\right)=\frac{A_{\mathrm{sample}}-A_{O}}{A_{\mathrm{control}}-A_{O}}\times 100\% \end{array}$$

where A_O_, A_sample_, and A_control_ are the absorbance values of the blank control group, the sample group, and the negative control group, respectively.

### 2.8. Measurement of Intracellular ROS

RAW264.7 macrophages were treated with LPS (1 µg/mL) to establish an inflammatory cell model. RAW264.7 cells were cultured until they were adherent to the wall and then the following medium was added (50 µg/mL CSA-CXB): (i) control group: complete medium only; (ii) LPS group: complete medium with LPS; (iii) LPS + CXB group: complete medium with LPS and CXB; (iv) LPS + CSA group: complete medium with LPS and CSA; (v) LPS + CSA-CXB group: complete medium containing LPS and CSA-CXB; and (vi) LPS + CSA + CXB group: complete medium containing LPS and a physical mixture of CSA and CXB.

Following a 24 h incubation period, the culture medium was removed and washed three times with PBS. Subsequently, 1 mL of 2′,7′-Dichlorodihydrofluorescein diacetate (DCFH-DA) was introduced, and the incubation was continued at 37 °C for 30 min. DCFH-DA that did not enter the cells was removed with PBS. RAW264.7 macrophages were gently scraped off and collected by centrifugation in Ep tubes at 1500 rpm for 3 min. Before flow cytometry analysis, 100 µL of PBS was added to each sample.

### 2.9. Determination of Anti-Inflammatory Effect In Vitro

RAW264.7 cells were cultured as described above (Section 2.8). After a 24 h incubation period, the cells were subjected to a 3 min centrifugation at 1000 rpm. Subsequently, the supernatant was centrifuged at 12,000 rpm for 15 min for removing cellular debris. The level of NO was measured using the NO assay kits, and ELISA kits were used to assess the levels of inflammatory cytokines (TNF-α and IL-6).

### 2.10. Western Blot Analysis

Western blotting analysis was conducted to assess the levels of COX-2 protein expression. RAW264.7 cells were cultured in medium as described above (Section 2.8) and then lysed by cell lysis buffer (RIPA:PMSF = 99:1). Each sample’s protein concentration was quantified and calculated with the BCA protein assay kit. The quantified sample was mixed with the loading buffer and subsequently underwent immunoblotting analysis. Ultimately, the membranes were photographed and observed with a Gel Imaging System (Image Lab Software Version 5.2.1).

### 2.11. Evaluation of Anti-Colitis Effect In Vivo

Female C57BL/6 mice (6 weeks, 18–20 g) were obtained from Gempharmatech Co., Ltd. (Nanjing, China). All animal procedures were carried out following the protocol approved by the Animal Study Committee of Jiangnan University with license no. JN. No20230830b0561024[354] and an approval date of 30 August 2023. 

The acute colitis mice model was established by feeding them 3% DSS continuously for 7 days and then changing to normal distilled water. The 36 mice were randomly separated into 6 groups, and each group was provided with a standard diet and treated as follows every other day: (i) control group: healthy mice fed with normal distilled water; (ii) DSS group: DDS-induced mice were injected with 200 µL of PBS in the tail vein; (iii) DSS + CXB group: DDS-induced mice were gavaged 10 mg/kg/d CXB; (iv) DSS + CSA group: DDS-induced mice were gavaged 80 mg/kg/d CSA; (v) DSS + CSA-CXB group: DDS-induced mice were injected with 200 µL of CSA-CXB (equivalent to 10 mg/kg/d CXB) in the tail vein; and (vi) DSS + CSA + CXB group: DDS-induced mice were injected with 200 µL of CSA and CXB mixture (equivalent to 10 mg/kg/d CXB) in the tail vein.

The experiment lasted for 9 days, and all mice were sacrificed on the 10th day. Then, the colon lengths were measured and photographed.

### 2.12. Bioimaging of Colon-Targeting Effect

Normal mice and DSS-induced mice were injected with 200 µL of Cy5 labeled CSA-CXB (Cy5 dose: 2.0 mg/kg), respectively. The mice were sacrificed 6 h later, and the colon tissues were isolated and imaged on an imaging system.

### 2.13. In Vivo Inflammatory Cytokine Test

C57BL/6 mice’s blood was centrifuged at 1000× *g* for 10 min after being placed at 4 °C for 30 min. The mouse colon was cut into small pieces and washed twice with PBS. Subsequently, it was lysed by cell lysis buffer (RIPA:PMSF = 99:1) and centrifuged at 10,000× *g* at 4 °C for 5 min. TNF-α and IL-6 levels in the supernatant (serum and colon) were measured using ELISA kits.

### 2.14. Statistical Analysis

Data were tested using an unpaired *t*-test when two groups were compared and was tested by analysis of variance (ANOVA) and Tukey post hoc test when more than two groups were compared. Statistical analysis was performed using R (version 3.6.1) and GraphPad Prism (version 8.2.1). All experimental data were represented as mean ± standard deviation (SD) and performed using one-way analysis of variance (ANOVA). Statistically significant difference means *p* < 0.05 (* *p* < 0.05, ** *p* < 0.01, *** *p* <0.001).

## 3. Results and Discussion

### 3.1. Characterization of CSA-CXB Polymers

**Figure 2:**
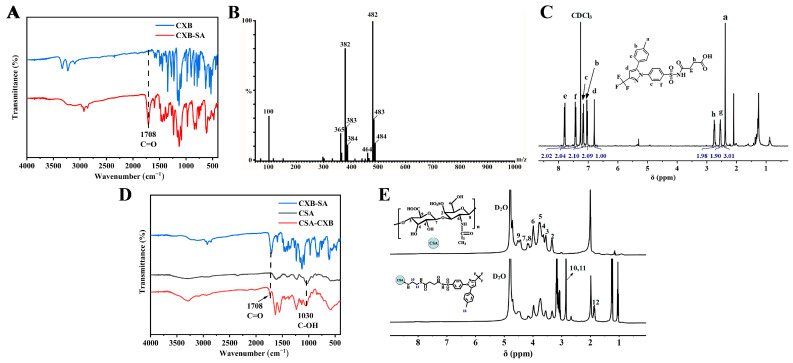
Characterization of CSA-CXB polymers. FTIR spectra (**A**), mass spectrometry result (**B**), and ^1^H NMR spectra (**C**) of CXB-SA. FTIR spectra (**D**) and ^1^H NMR spectra (**E**) of CSA-CXB polymers.

The polymorphic form of CXB was first measured by PXRD. As shown in Figure 1A, the PXRD pattern conclusively demonstrated that CXB used in this work corresponded to Form III, which aligns with the crystallographic data reported in the literature [37,38]. Then, CXB was modified by SA, and then conjugated to CSA backbone using ethylenediamine as a linker to synthesize CSA-CXB polymers (Figure 1B,C). As shown from the FTIR spectrum, CXB-SA showed a distinct carbonyl peak at 1708 cm^−1^ compared to CXB (Figure 2A). The molar mass of CXB-SA is 482 g/mol, which is consistent with the MS results (Figure 2B), further confirming the successful synthesis of the CXB-SA intermediate. Meanwhile, the peak representing SA at 2.53 and 2.67 ppm appeared in the ^1^H NMR spectrum of CXB-SA (Figure 2C), indicating that CSA-SA contains the structures of SA. Furthermore, the same characteristic peaks of CXB-SA and CSA were visible in the FTIR spectrum of CSA-CXB (Figure 2D). The ^1^H NMR spectrum of the CSA-CXB polymer displayed new peaks at 2.80, 2.66, and 1.83 ppm compared to that of CSA, indicating the presence of CXB-SA (Figure 2E). The above results demonstrated the successful synthesis of the CXB-SA precursor and CSA-CXB polymers. The grafting ratio was calculated as the amount of CXB per 100 sugar residues in CSA [39]. As shown in Figure 2E, based on the peak area ratio of the characteristic peak of CSA (δ = 3.31 ppm) to the benzyl group of CXB (δ = 1.83 ppm) in the ^1^H NMR spectrum of CSA-CXB, the grafting rate of CSA-CXB was obtained as 18.7%.

### 3.2. Characterization of CSA-CXB Nanoparticles

**Figure 3:**
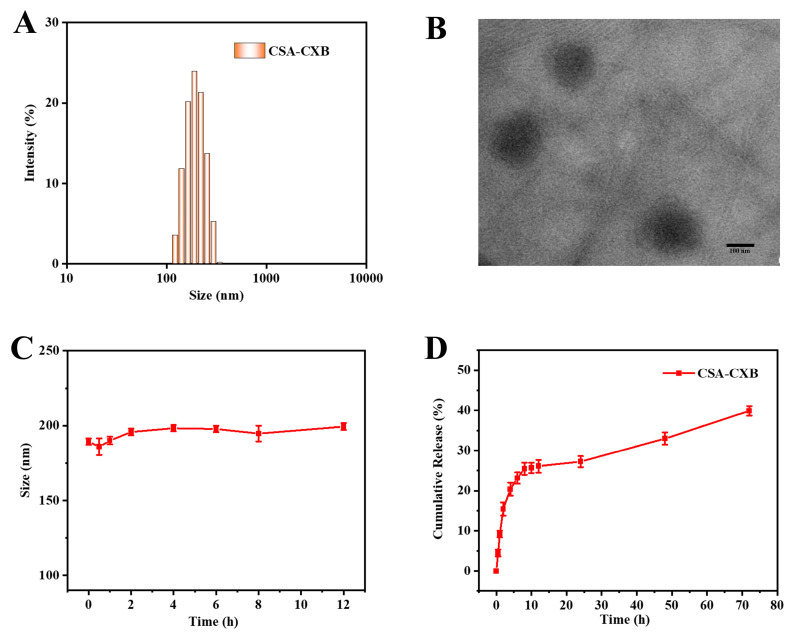
Characterization and in vitro drug release behavior of CSA-CXB nanoprodrug. Size distribution (**A**) and TEM image (scale bar: 100 nm) (**B**) of CSA-CXB nanoprodrug. Stability of CSA-CXB in serum (**C**). Release of CXB from CSA-CXB polymeric nanoprodrug in PBS at 37 °C (**D**).

The amphiphilic CSA-CXB polymer can self-assemble into a nanoparticle structural prodrug in an aqueous environment. The particle size of the CSA-CXB nanoprodrug was 188.4 ± 2.2 nm (Figure 3A), and it showed uniform spherical shape when tested by TEM (Figure 3B). The zeta potential was −22.9 ± 0.1 mV, which can prevent the absorption of proteins in blood circulation. Therefore, FBS was used to simulate the blood environment to investigate whether the CSA-CXB nanoprodrug could remain stable in the blood. As shown from Figure 3C, CSA-CXB showed no significant changes in particle size after incubating in the FBS environment for 12 h, which demonstrated that the nanoprodrug was stable in blood.

### 3.3. In Vitro Drug Release Behavior

Drug release of CXB from the CSA-CXB nanoprodrug in vitro was investigated in PBS (pH 7.4, 37 °C). CXB exhibited a rapid release during the initial 10 h period, reaching a cumulative release rate of 25%, which was then followed by steady release behavior over 72 h (Figure 3D). The release rate of CSA-CXB may be related to the hydrolysis of the covalent bonds in the first stage. The covalent bonds close to the nanoparticle shell easily encounter the release medium, resulting in rapid drug release. Subsequently, the drug release rate gradually slowed down as the internal covalent bonds of CSA-CXB were involved in the hydrolysis reaction. Moreover, the released drug needs to travel longer distances to enter the release medium, and the polysaccharide polymer may also affect the drug diffusion behavior in the process.

### 3.4. Biocompatibility Assessment of CSA-CXB

**Figure 4:**
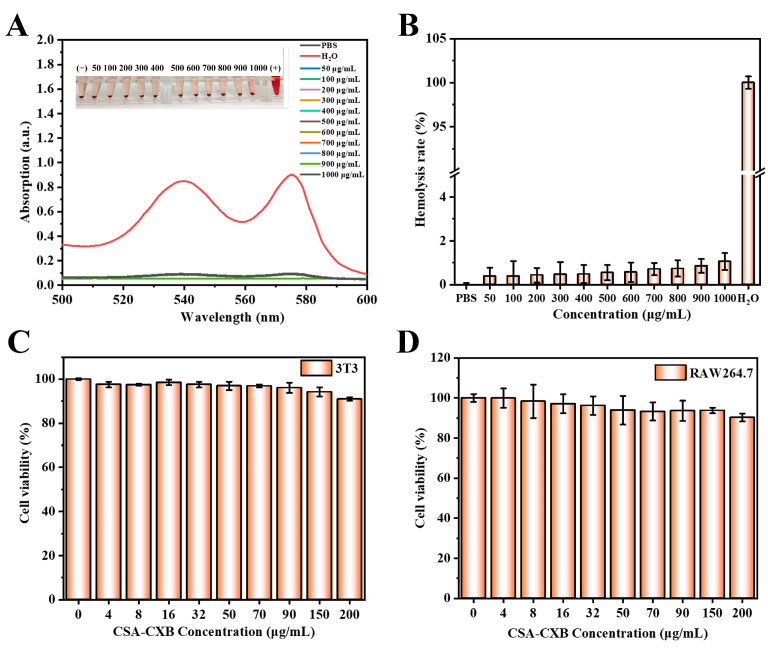
Ultraviolet absorption spectra of CSA-CXB at different concentrations (**A**) and hemolysis rates (**B**) of red blood cells incubated with different concentrations of CSA-CXB. In vitro cytotoxicity of CSA-CXB against 3T3 cells (**C**) and RAW264.7 cells (**D**).

Biocompatibility is a critical feature for polymer-based drug delivery system applications. The preliminary biocompatibility of CSA-CXB was assessed by hemolysis and cytotoxicity tests. There was no significant hemolysis found at different concentrations of CSA-CXB (Figure 4A,B), and the hemolysis rates were less than 5%. The results indicated that CSA-CXB did not induce red blood cells to hemolyze. In addition, the cytotoxicity of CSA-CXB was evaluated by MTT assay. Following a 24 h incubation period with CSA-CXB at different concentrations (0 to 200 μg/mL), both 3T3 and RAW264.7 cells showed high viability of more than 90% (Figure 4C,D). This result revealed that CSA-CXB had a good safety profile in normal and immune cells, indicating the nanoprodrug had the potential to be suitable for in vivo applications.

### 3.5. Anti-Inflammatory and Antioxidant Activities

**Figure 5:**
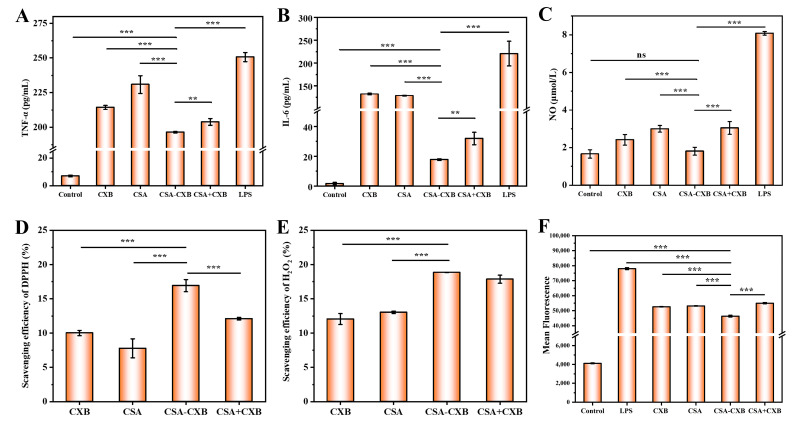
In vitro anti-inflammatory and antioxidant activities of different formulations. TNF-α level (**A**), IL-6 level (**B**), NO level (**C**), DPPH (**D**), H_2_O_2_ (**E**) scavenger capacity, and ROS level (**F**) of different formulations. (** *p* < 0.01, *** *p* < 0.001, ns means not significant).

The pathogenesis of UC is associated with a complex interplay between inflammation and oxidative stress. Inflammation can lead to the production of substantial ROS, and oxidative stress caused by the accumulated ROS can induce oxidative damage and amplify the inflammatory response, forming a vicious cycle that leads to ongoing intestinal damage [40]. The disruption of the intestinal barrier activates immune cells to produce high concentrations of inflammatory cytokines, which in turn promote the inflammatory response. So, the in vitro anti-inflammatory properties of the CSA-CXB nanoprodrug were assessed by the expression of TNF-α and IL-6. The findings indicated that LPS treatment substantially increased inflammatory cytokine expression, while CSA-CXB showed the strongest inhibitory effect compared to the other groups on TNF-α (Figure 5A) and IL-6 expression (Figure 5B). NO also plays an important part in the inflammatory processes, and there have been many reports of NOS increase and NO production in UC patients [7,41]. As shown in Figure 5C, CSA-CXB significantly decreased the expression level of NO. This is consistent with the results of TNF-α and IL-6 test, proving CSA-CXB’s potential anti-inflammatory capacity.

The in vitro antioxidant activity of the CSA-CXB nanoprodrug was evaluated by measuring DPPH free radicals and H_2_O_2_. As shown from Figure 5D, the scavenging efficiency of DPPH free radicals by CSA-CXB reached 16.92%, which was markedly higher than the free CSA, CXB, and CSA + CXB (*** *p* < 0.001). Similarly, CSA-CXB exhibited a scavenging efficiency of about 19% in the H_2_O_2_ assay, which was also higher than the free CSA and CXB (*** *p* < 0.001) (Figure 5E). However, there was no notable distinction between the CSA-CXB nanoprodrug and the CSA + CXB physical mixture. This may be because CXB released from CSA-CXB mainly exerted an antioxidant effect, and the low water solubility of CXB limited its effectiveness in H_2_O_2_ scavenging. Moreover, the intracellular ROS content which was detected by DCFH-DA fluorescent probe was investigated to further assess the antioxidant abilities of CSA-CXB. As shown in Figure 5F, CSA-CXB significantly reduced the intracellular ROS level (*** *p* < 0.001) among the groups. These results revealed that the CSA-CXB nanoprodrug showed good anti-inflammatory and antioxidant activity, with the potential to treat and inhibit UC.

### 3.6. Inhibitory Effect of COX-2 Protein Expression

**Figure 6:**
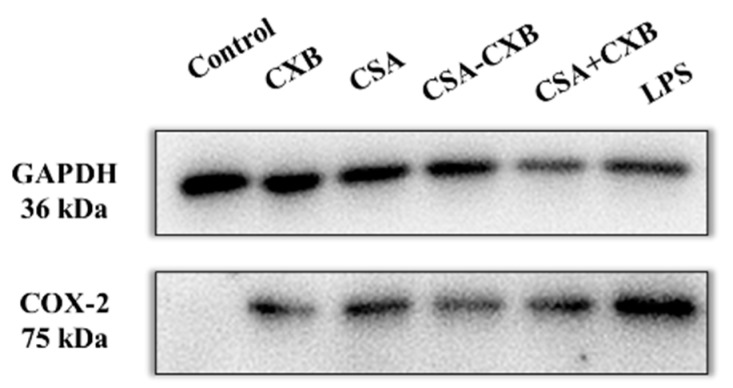
Expression of COX-2 protein detected by Western blotting.

In UC patients, COX-2 is shown to be highly expressed in the colonic mucosa [42]. Inflammation can stimulate the generation of the COX-2 protein, causing inflammation, edema, and pain, and increasing the severity of UC. CXB selectively inhibits COX-2 expression to reduce prostaglandin E2 synthesis, which achieves anti-inflammatory and analgesic effects. Additionally, it was reported that CSA may also mitigate inflammatory responses by decreasing COX-2 expression [43]. Therefore, COX-2 protein level was detected in inflammatory cells treated with different formulations. The results of the Western blot analysis (Figure 6) demonstrated that the COX-2 protein was abundantly expressed following LPS stimulation, but free CXB and CSA, CSA + CXB mixture, and CSA-CXB reduced the COX-2 protein expression to some extent. Among them, the protein band of CSA-CXB was lightest, suggesting that the nanoprodrug significantly decreased the expression level of the COX-2 protein.

### 3.7. In Vivo Therapeutic Effect of CSA-CXB

**Figure 7:**
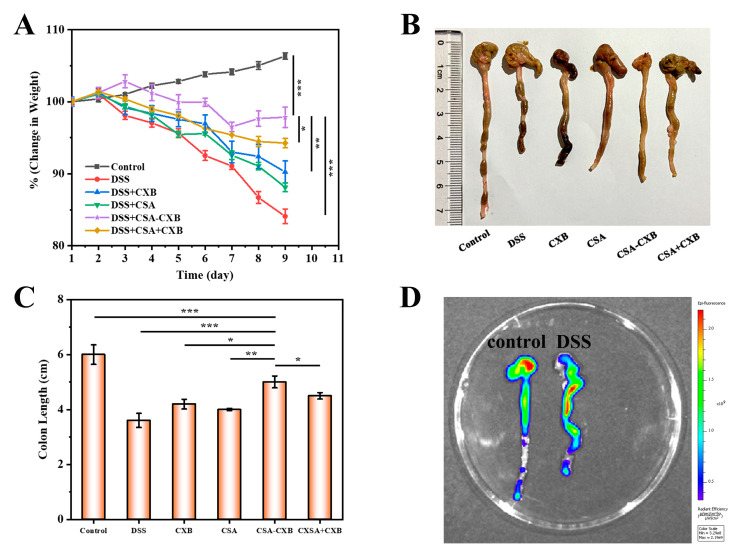
Effects of CSA-CXB on UC mice. Body weight changes (**A**), colon images (**B**), and length (**C**) of different formulations. In vivo colon targeting imaging of CSA-CXB nanoprodrug in healthy (left) and DDS-induced mice (right) (**D**). (* *p* < 0.05, ** *p* < 0.01, *** *p* < 0.001).

The body weight and colon length of the mice were recorded to assess the in vivo therapeutic efficacy of the CSA-CXB nanoprodrug on UC. As shown from Figure 7A, the healthy mice of the control group exhibited a sustained weight increase, while the DSS-treated mice demonstrated a persistent weight loss, indicating the successful establishment of the UC animal model. Compared with CXB, CSA, and CXB + CSA, the administration of CXB-CSA significantly improved body weight loss. 

Furthermore, as is consistent with the body weight changes, the DSS-treated mice have considerably shorter colons than the healthy mice, and CXB-CSA significantly inhibited the reduction in colon length compared to other groups (Figure 7B,C). These phenomena might be caused by CSA-CXB’s synergistic anti-inflammatory effects. Additionally, CSA-CXB could accumulate at colitis regions through the EPR and CD44 active targeting effects. Overall, these findings demonstrated that CXB-CSA had a potential therapeutic effect on UC. To further evaluate CSA-CXB’s potential for in vivo targeting, an imaging system was employed to observe the colonic tissue following the injection of the Cy5-labeled CSA-CXB nanoprodrug. Compared to the healthy mice, CSA-CXB showed a higher concentration at the UC colonic site (Figure 7D). This result confirmed that CSA-CXB accumulated effectively in the colon by CD44 receptor-mediated active targeting of CSA.

### 3.8. Investigation of Anti-Inflammatory Mechanism In Vivo

**Figure 8:**
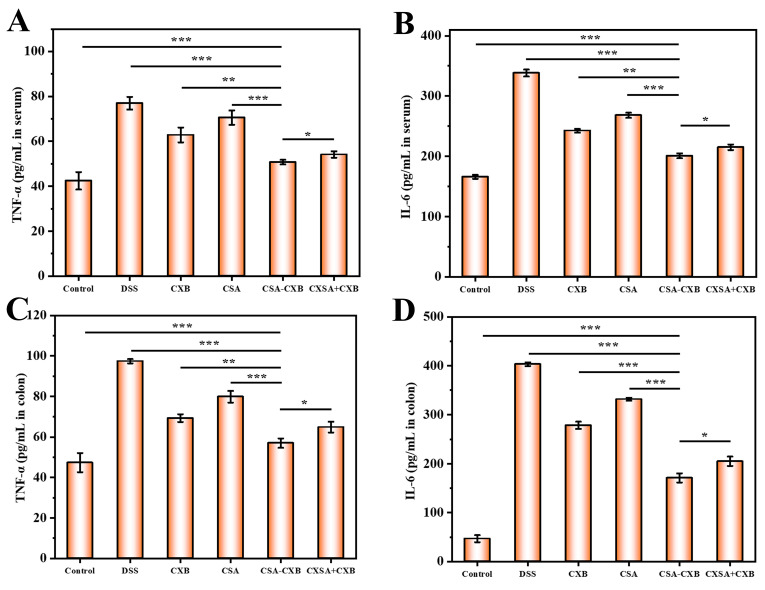
In vivo inflammatory cytokines levels of TNF-α and IL-6 in serum (**A**,**B**) and colon (**C**,**D**). (* *p* < 0.05, ** *p* < 0.01, *** *p* < 0.001).

To assess the anti-inflammatory mechanism in vivo of the CSA-CXB nanoprodrug, inflammatory cytokine levels in the serum and colon were measured. As shown from Figure 8, the expressions of TNF-α and IL-6 were markedly raised in the serum and colon after DSS treatment. However, after being treated with CXB, CSA, CSA-CXB, and CSA + CXB, the expressions of TNF-α and IL-6 were effectively inhibited in the serum and colon, suggesting that CXB and CSA had some anti-inflammatory effects. It is worth noting that CSA-CXB showed the greatest effect on reducing the pro-inflammatory cytokines, which is in line with the outcomes of the in vivo treatment.

## 4. Conclusions

In this work, a new CSA-CXB nanoprodrug delivery system was developed to improve the solubility and bioavailability of CXB using polysaccharide polymers. The prepared CSA-CXB self-assembled into nanoparticles and exhibited good antioxidant and anti-inflammatory effects. In vivo studies revealed that CSA-CXB could target the colon site and enhance CXB accumulation, resulting in alleviating the symptoms of DSS-induced UC mice. Consequently, the CSA-CXB polymeric nanoprodrug could be considered a potential anti-inflammatory therapy for UC.

## Data Availability

The original contributions presented in this study are included in the article.

## References

[1] Le Berre C., Honap S., Peyrin-Biroulet L. (2023). Ulcerative Colitis. Lancet.

[2] Wang J., Mao T., Zhou H., Jiang X., Zhao Z., Zhang X. (2023). Global Trends and Hotspots of Ulcerative Colitis Based on Bibliometric and Visual Analysis from 1993 to 2022. Medicine.

[3] Deng J., Xian D., Cai X., Liao S., Lei S., Han F., An Y., He Q., Quan G., Wu C. (2023). Surface-Engineered Vanadium Carbide MXenzyme for Anti-Inflammation and Photoenhanced Antitumor Therapy of Colon Diseases. Adv. Funct. Mater..

[4] Grivennikov S.I., Greten F.R., Karin M. (2010). Immunity, Inflammation, and Cancer. Cell.

[5] Zhang M., Li X., Zhang Q., Yang J., Liu G. (2023). Roles of Macrophages on Ulcerative Colitis and Colitis-Associated Colorectal Cancer. Front. Immunol..

[6] Porter R.J., Kalla R., Ho G.-T. (2020). Ulcerative Colitis: Recent Advances in the Understanding of Disease Pathogenesis. F1000Research.

[7] Kimura H., Miura S., Shigematsu T., Ohkubo N., Tsuzuki Y., Kurose I., Higuchi H., Akiba Y., Hokari R., Hirokawa M. (1997). Increased Nitric Oxide Production and Inducible Nitric Oxide Synthase Activity in Colonic Mucosa of Patients with Active Ulcerative Colitis and Crohn’s Disease. Dig. Dis. Sci..

[8] Gajendran M., Loganathan P., Jimenez G., Catinella A.P., Ng N., Umapathy C., Ziade N., Hashash J.G. (2019). A Comprehensive Review and Update on Ulcerative Colitis. Dis.-A-Mon..

[9] Aslam N., Lo S.W., Sikafi R., Barnes T., Segal J., Smith P.J., Limdi J.K. (2022). A Review of the Therapeutic Management of Ulcerative Colitis. Ther. Adv. Gastroenterol..

[10] McCormack P.L. (2011). Celecoxib: A Review of Its Use for Symptomatic Relief in the Treatment of Osteoarthritis, Rheumatoid Arthritis and Ankylosing Spondylitis. Drugs.

[11] Lin X., Sun Q., Zhou L., He M., Dong X., Lai M., Liu M., Su Y., Jia C., Han Z. (2018). Colonic Epithelial mTORC1 Promotes Ulcerative Colitis through COX-2-Mediated Th17 Responses. Mucosal Immunol..

[12] Li Y., Ma M., Wang X., Li J., Fang Z., Li J., Yang B., Lu Y., Xu X., Li Y. (2024). Celecoxib Alleviates the DSS-Induced Ulcerative Colitis in Mice by Enhancing Intestinal Barrier Function, Inhibiting Ferroptosis and Suppressing Apoptosis. Immunopharmacol. Immunotoxicol..

[13] Song W.H., Yeom D.W., Lee D.H., Lee K.M., Yoo H.J., Chae B.R., Song S.H., Choi Y.W. (2014). In Situ Intestinal Permeability and in Vivo Oral Bioavailability of Celecoxib in Supersaturating Self-Emulsifying Drug Delivery System. Arch. Pharm. Res..

[14] Abdul Khader A.H.S., Singh M. (2024). Celecoxib-Induced Acute Generalized Exanthematous Pustulosis: Uncommon and under-Recognized Side Effect. EXCLI J..

[15] Cruz J.V., Rosa J.M.C., Kimani N.M., Giuliatti S., Dos Santos C.B.R. (2022). The Role of Celecoxib as a Potential Inhibitor in the Treatment ofInflammatory Diseases—A Review. CMC.

[16] Barclay T.G., Day C.M., Petrovsky N., Garg S. (2019). Review of Polysaccharide Particle-Based Functional Drug Delivery. Carbohydr. Polym..

[17] Wu Q., Hu Y., Yu B., Hu H., Xu F.-J. (2023). Polysaccharide-Based Tumor Microenvironment-Responsive Drug Delivery Systems for Cancer Therapy. J. Control. Release.

[18] Mohammed A.S.A., Naveed M., Jost N. (2021). Polysaccharides; Classification, Chemical Properties, and Future Perspective Applications in Fields of Pharmacology and Biological Medicine (A Review of Current Applications and Upcoming Potentialities). J. Polym. Environ..

[19] Sood A., Gupta A., Agrawal G. (2021). Recent Advances in Polysaccharides Based Biomaterials for Drug Delivery and Tissue Engineering Applications. Carbohydr. Polym. Technol. Appl..

[20] Mohan T., Kleinschek K.S., Kargl R. (2022). Polysaccharide Peptide Conjugates: Chemistry, Properties and Applications. Carbohydr. Polym..

[21] Wang J., Zhao W., Chen H., Qin A., Zhu P. (2017). Anti-Tumor Study of Chondroitin Sulfate-Methotrexate Nanogels. Nanoscale Res. Lett..

[22] Iovu M., Dumais G., du Souich P. (2008). Anti-Inflammatory Activity of Chondroitin Sulfate. Osteoarthr. Cartil..

[23] du Souich P., García A.G., Vergés J., Montell E. (2009). Immunomodulatory and Anti-Inflammatory Effects of Chondroitin Sulphate. J. Cell. Mol. Med..

[24] Xie Y., Xu W., Jin Z., Zhao K. (2023). Chondroitin Sulfate Functionalized Palmitic Acid and Cysteine Cografted-Quaternized Chitosan for CD44 and Gut Microbiota Dual-Targeted Delivery of Curcumin. Mater. Today Bio.

[25] Rosenberg W.M.C., Jackson D.G., Trowell J.M., Bell J.I., Rosenberg W.M.C., Prince C., Chapman R.W., Trowell J.M., Jewel D.P., Kaklamanis L. (1995). Increased Expression of CD44v6 and CD44v3 in Ulcerative Colitis but Not Colonic Crohn’s Disease. Lancet.

[26] Harris A.J., Dean D., Burge S., Wojnarowska F. (1997). Changes in CD44 Isoform Expression during Inflammatory Skin Disease. Clin. Exp. Dermatol..

[27] Azimijou N., Karimi-Soflou R., Karkhaneh A. (2024). CD44 Targeted-Chondroitin Sulfate Nanoparticles: Fine-Tuning Hydrophobic Groups to Enhance in Vitro pH-Responsiveness and in Vivo Efficacy for Advanced Breast Cancer Treatment. Biomater. Adv..

[28] Kesharwani P., Chadar R., Sheikh A., Rizg W.Y., Safhi A.Y. (2022). CD44-Targeted Nanocarrier for Cancer Therapy. Front. Pharmacol..

[29] Li M., Sun J., Zhang W., Zhao Y., Zhang S., Zhang S. (2021). Drug Delivery Systems Based on CD44-Targeted Glycosaminoglycans for Cancer Therapy. Carbohydr. Polym..

[30] Li Y., Hou H., Liu Z., Tang W., Wang J., Lu L., Fu J., Gao D., Zhao F., Gao X. (2023). CD44 Targeting Nanodrug Based on Chondroitin Sulfate for Melanoma Therapy by Inducing Mitochondrial Apoptosis Pathways. Carbohydr. Polym..

[31] Liu P., Chen N., Yan L., Gao F., Ji D., Zhang S., Zhang L., Li Y., Xiao Y. (2019). Preparation, Characterisation and in Vitro and in Vivo Evaluation of CD44-Targeted Chondroitin Sulphate-Conjugated Doxorubicin PLGA Nanoparticles. Carbohydr. Polym..

[32] Brusini R., Varna M., Couvreur P. (2020). Advanced Nanomedicines for the Treatment of Inflammatory Diseases. Adv. Drug Deliv. Rev..

[33] Crielaard B.J., Lammers T., Schiffelers R.M., Storm G. (2012). Drug Targeting Systems for Inflammatory Disease: One for All, All for One. J. Control. Release.

[34] Gao J., Li J., Luo Z., Wang H., Ma Z. (2024). Nanoparticle-Based Drug Delivery Systems for Inflammatory Bowel Disease Treatment. DDDT.

[35] Lee Y., Kim J., Kim H., Kang S., Yoon J.-H., Kim D.-D., Kim Y.M., Jung Y. (2012). N-Succinylaspart-1-Yl Celecoxib Is a Potential Colon-Specific Prodrug of Celecoxib with Improved Therapeutic Properties. J. Pharm. Sci..

[36] Seetharaman G., Kallar A.R., Vijayan V.M., Muthu J., Selvam S. (2017). Design, Preparation and Characterization of pH-Responsive Prodrug Micelles with Hydrolyzable Anhydride Linkages for Controlled Drug Delivery. J. Colloid Interface Sci..

[37] Dr. Reddy’s Laboratories Ltd (2011). Process for Preparation of Celecoxib Crystalline Form.

[38] Ferro L.J., Miyake P.S. (2009). Polymorphic Crystalline Forms of Celecoxib.

[39] Liu H., Wu S., Yu J., Fan D., Ren J., Zhang L., Zhao J. (2017). Reduction-Sensitive Micelles Self-Assembled from Amphiphilic Chondroitin Sulfate A-Deoxycholic Acid Conjugate for Triggered Release of Doxorubicin. Mater. Sci. Eng. C.

[40] Grisham M.B. (1994). Oxidants and Free Radicals in Inflammatory Bowel Disease. Lancet.

[41] Middleton S.J., Shorthouse M., Hunter J.O. (1993). Increased Nitric Oxide Synthesis in Ulcerative Colitis. Lancet.

[42] Singer I.I., Kawka D.W., Schloemann S., Tessner T., Riehl T., Stenson W.F. (1998). Cyclooxygenase 2 Is Induced in Colonic Epithelial Cells in Inflammatory Bowel Disease. Gastroenterology.

[43] Cañas N., Gorina R., Planas A.M., Vergés J., Montell E., García A.G., López M.G. (2010). Chondroitin Sulfate Inhibits Lipopolysaccharide-Induced Inflammation in Rat Astrocytes by Preventing Nuclear Factor Kappa B Activation. Neuroscience.

